# Testing the impact of a single nucleotide polymorphism in a *Plasmodium berghei* ApiAP2 transcription factor on experimental cerebral malaria in mice

**DOI:** 10.1038/s41598-020-70617-7

**Published:** 2020-08-12

**Authors:** Munir Akkaya, Abhisheka Bansal, Patrick W. Sheehan, Mirna Pena, Clare K. Cimperman, Chen Feng Qi, Takele Yazew, Thomas D. Otto, Oliver Billker, Louis H. Miller, Susan K. Pierce

**Affiliations:** 1 Laboratory of Immunogenetics, National Institute of Allergy and Infectious Diseases, National Institutes of Health, 5625 Fishers Lane, Room 4S04, Rockville, MD USA; 2 Laboratory of Malaria and Vector Research, National Institute of Allergy and Infectious Diseases, National Institutes of Health, Rockville, MD USA; 3 Institute of Infection, Immunity and Inflammation, University of Glasgow, Glasgow, UK; 4 Laboratory for Molecular Infection Medicine Sweden and Molecular Biology Department, Umea University, Umea, Sweden; 5 Present Address: School of Life Sciences, Jawaharlal Nehru University, New Delhi, India; 6 Present Address: Department of Neurology, Washington University School of Medicine in St. Louis, St. Louis, MO USA; 7 Present Address: Department of Microbiology, University of Pennsylvania, Philadelphia, PA USA; 8 Present Address: Department of Veterinary Medicine, College of Agriculture and Natural Resources, University of Maryland, College Park, MD USA

**Keywords:** Parasite genetics, Parasite genomics, Parasite host response, Parasite immune evasion, Parasitic infection

## Abstract

Cerebral malaria (CM) is the deadliest form of severe *Plasmodium* infections. Currently, we have limited understanding of the mechanisms by which *Plasmodium* parasites induce CM. The mouse model of CM, experimental CM (ECM), induced by infection with the rodent parasite, *Plasmodium berghei* ANKA (*Pb*ANKA) has been extensively used to study the pathophysiology of CM. Recent genomic analyses revealed that the coding regions of *Pb*ANKA and the closely related *Plasmodium berghei* NK65 (*Pb*NK65), that does not cause ECM, differ in only 21 single nucleotide polymorphysims (SNPs). Thus, the SNP-containing genes might contribute to the pathogenesis of ECM. Although the majority of these SNPs are located in genes of unknown function, one SNP is located in the DNA binding site of a member of the *Plasmodium* ApiAP2 transcription factor family, that we recently showed functions as a virulence factor alternating the host’s immune response to the parasite. Here, we investigated the impact of this SNP on the development of ECM. Our results using CRISPR-Cas9 engineered parasites indicate that despite its immune modulatory function, the SNP is neither necessary nor sufficient to induce ECM and thus cannot account for parasite strain-specific differences in ECM phenotypes.

## Introduction

Malaria is a global health problem accounting for over 200 million cases each year worldwide and nearly 450,000 deaths in Africa alone, the majority of which are young children^1,2^. Malaria is caused by mosquito-borne parasites belonging to genus *Plasmodium* that have complex life cycles involving two hosts^3^. The outcome of the *Plasmodium* infection can vary from asymptomatic infections, to mild febrile disease, to severe malaria, the most deadly form of which is cerebral malaria (CM)^3–7^. Approximately 1–2% of children with malaria develop CM that is accompanied by sequestration of infected RBCs (iRBCs) in the brain vasculature, blood brain barrier dysfunction, brain swelling and ultimately herniation of the brain stem and death^6–8^. CM mortality is high, 15–25%, and tragically, approximately 25% of children who recover from CM suffer from long-term neurological sequalae, including cognitive, vision and hearing impairments^7^.

An important tool for the study of CM is the mouse model of CM, namely experimental CM (ECM). ECM is induced by infection of susceptible mouse strains such as C57BL/6 with the ECM-causing rodent *Plasmodium* strain, *Pb*ANKA that recapitulates many of the features of CM in children both clinically and pathologically^7–14^. *Pb*ANKA infection results in the rapid progression of disease, leading to development of as ataxia, paralysis and coma accompanied by brain hemorrhages, iRBC sequestration in brain vessels, edema, and if left untreated, within 5–7 days post infection, death, likely by neuronal cell death in the brain stem^11,12^. Repeated high resolution brain MRI monitoring of *Pb*ANKA-infected mice showed radiological findings indicating vasogenic edema and blood brain barrier (BBB) disruption similar to cerebral pathology described by MRI in children with CM^13^. Mice treated with anti-malarials when neurological symptoms first appear, as is the standard of care for children, show long-term cognitive dysfunction^13^. In the mouse model CD8^+^ T cells that migrate to and sequester in the brain vasculature have been demonstrated to be directly responsible for ECM mortality^15^. Because only a few histopathological studies reported leukocyte accumulation in the brains of children who died of CM, the ECM model was deemed questionable^16^. However, a recent demonstration of CD8^+^ T cells in contact with the brain endothelium in children who died of CM but not of other causes, provided strong evidence that the mouse model accurately reflects the human disease pathology^17^. Therefore, ECM is an important animal model that has the potential to increase field’s understanding of this deadly disease.

In contrast, a highly related strain to *Pb*ANKA, *Pb*NK65, causes severe anemia in the absence of any detectable brain pathology^18,19^. A comparison of high coverage genomic sequences of *Pb*ANKA and *Pb*NK65 revealed that these two strains differ by only 21 single nucleotide polymorphisms (SNPs) in their coding regions (Table 1)^20^. Thus, remarkably, a small number of SNPs may account for the dramatically different disease outcomes of infection with *Pb*ANKA versus *Pb*NK65. The majority of the genes containing SNPs are of unknown function, however, two SNPs were identified in genes encoding proteins belonging to ApiAP2 transcriptional factor (TF) family namely PBANKA_0112100 and PBANKA_1415700. The SNP in PBANKA_1415700 was located immediately before the stop codon and hence unlikely to induce structural alterations that would lead to functional differences in the expressed ApiAP2 (Table 1). On the other hand, the SNP in PBANKA_0112100 was located in the predicted DNA-binding domain of ApiAP2 resulting in a substitution of a Serine (S) to Phenylalanine (F) at amino acid 1823 in the expressed protein, two biochemically distinct amino acids. Therefore, this SNP had the potential to influence the function of ApiAP2.

**Table 1:** Locations of all 21 SNPs that are different between the coding regions of ECM causing *Pb*ANKA and non-ECM causing *Pb*NK65 parasites.

SNP	Gene ID	Product	Length	Position of mutation	Base in ANKA	Base in NK65	ANKA sequence	NK65 sequence	aa ANKA	aa NK65
1	PBANKA_030400	Conserved Plasmodium protein—unknown function	5,934	3,239	G	A	AGA	AAA	R	K
2	PBANKA_144660	Conserved Plasmodium protein—unknown function	3,093	2,204	T	A	ATA	AAA	I	K
3	PBANKA_0112100	Transcription factor with AP2 domain(s)	7,737	5,468	T	C	TTT	TCT	F	S
4	PBANKA_090710	Inner membrane complex protein 1b	1,608	364	A	G	ACC	GCC	T	A
5	PBANKA_122210	Regulator of chromosome condensation, putative	1,953	1,465	T	G	TAT	GAT	Y	D
6	PBANKA_134180	Conserved Plasmodium protein—unknown function	12,030	9,202	A	T	AAT	TAT	N	Y
7	PBANKA_140230	Conserved Plasmodium protein—unknown function	24,729	11,948	C	T	TGC	TAC	C	Y
8	PBANKA_141570	Transcription factor with AP2 domain(s)	7,464	7,461	G	A	ATG	ATA	M	I
9	PBANKA_083100	Merozoite surface protein 1	5,376	4,096	A	G	AGA	GGA	R	G
10	PBANKA_144610	Amino acid transporter, putative	5,313	5,070	C	A	AAG	AAT	K	N
11	PBANKA_030600	6-cysteine protein	6,864	1,852	C	A	CCA	ACA	P	T
12	PBANKA_081770	RNA-binding protein musashi, putative	1,035	611	C	T	GGA	GAA	G	E
13	PBANKA_092040	Conserved Plasmodium protein—unknown function	5,709	2,691	A	T	AAT	AAA	N	K
14	PBANKA_010160	Conserved Plasmodium protein—unknown function	2,025	462	T	A	AAT	AAA	N	K
15	PBANKA_051520	MORN repeat-containing protein 1, putative	1,095	519	A	G	ATA	ATG	I	M
16	PBANKA_080220	Serine/threonine kinase, putative	3,873	2,611	C	T	GAT	AAT	D	N
17	PBANKA_082480	RNA-binding protein, putative	600	517	G	A	CTT	TTT	L	F
18	PBANKA_143400	Phosphate translocator, putative	1,440	161	C	T	AGA	AAA	R	K
19	PBANKA_141460	Inositol polyphosphate kinase, putative	3,129	1,888	A	G	ATA	GTA	I	V
20	PBANKA_133170	Zinc finger protein, putative	2,226	1,347	A	C	AGA	AGC	R	S

PBANKA_0112100 is an essential transcription factor and therefore no viable knock out could be generated^21^. Until recently it was a gene of unknown function which is expressed in the schizonts during the blood stage of *Plasmodium* life cycle^21^. However, our thorough genetic and functional analysis using this SNP in order to dissect out the function of this transcription factor revealed that the SNP in the DNA binding region of PBANKA_0112100 alters the expression of 46 *Plasmodium* genes. Among these 46 genes 39 belong to either BIR(22/46), fam-a (7/46), fam-b ( 8/46) or fam-c (2/46) gene families all of which are known to be involved in virulence and evasion related functions^22^. Based on these changes we have observed differences in host pathogen interaction including changes in protective immune responses against the parasite^22^. Despite these published findings whether or not this SNP alters the progression of ECM is not clear. Here we address a possible link between this polymorphism in ApiAP2 and the development of ECM.

## Results

### CRISPR-Cas9 gene editing strategy generated two viable mutant parasites

In order to test whether the SNP leading to non-synonymous S to F substitution in 1823^rd^ amino acid of the ApiAP2 TF family member PBANKA_0112100 plays a role in the development of ECM, we engineered two distinct transgenic parasites; *Pb*NK65^F^ that contained an S → F substitution and *Pb*ANKA^S^ that contained F → S substitution at position 1823 in the PbNK65 and PbANKA WT parasite backgrounds, respectively using CRISPR-Cas9 (Supplementary Fig. 1). A single plasmid (pYC) system as described by Zhang et al. for gene editing in *P*. *yoelii* was used to introduce the SNP at 1823 position in ApiAP2^23^. The pYC plasmid contains all the essential components of CRISPR-Cas9 that are required for successful genome editing. Our earlier and present work demonstrate successful use of pYC for genome editing of *P*. *berghei* in addition to its initial usage for *P*. *yoelii* genome engineering^23^. A guide region of 20 nucleotides was manually selected near amino acid position 1823 that followed the PAM motif (Supplementary Fig. 1A). The guide was cloned upstream of tracrRNA sequence in pYC to enable PyU6 promoter driven expression of gRNA:tracrRNA chimera^23^. The desired mutation was incorporated in the transgenic parasites along with shield mutations that were introduced in the modified locus to avoid recognition by the Cas9 endonuclease (Supplementary Fig. 1A). The desired and the shield mutations were part of synthetic gene sequence that was also encoded in pYC plasmid (see Supplementary Information). The incorporation of the desired substitution (F → S) in AP2 gene of PbANKA^S^ was confirmed by DNA sequencing (Supplementary Fig. 1B). We compared infections by the mutant parasites *Pb*NK65^F^ and *Pb*ANKA^S^ in C57BL/6 mice to infections with their WT counterparts *Pb*NK65^S^ and *Pb*ANKA^F^, respectively. Notably, we have recently functionally characterized the transgenic PbNK65^F^ parasites and found that the mutant AP2 is involved in mounting protective immune response in the infected host that is positively correlated with host survival in second challenge with either the WT or the mutant strain of PbNK65^22^.

**Figure 1:**
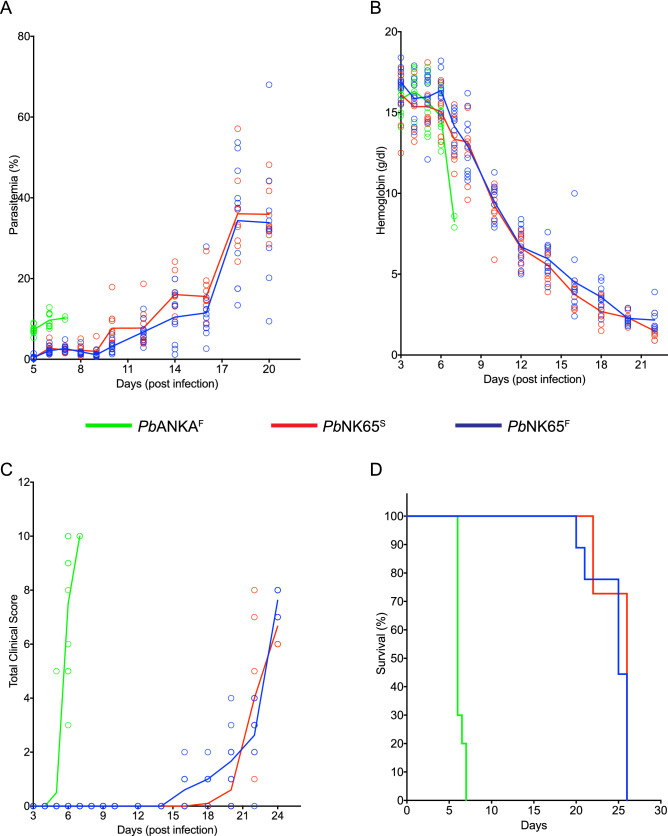
S1823F mutation in ApiAP2 TF of *Pb*NK65 does not alter the progression of infection. (**A**–**D**) C57BL/6 mice were inoculated intraperitoneally with WT *Pb*ANKA^F^-, WT *Pb*NK65^S^- and mutant *Pb*NK65 ^F^ iRBCs (10^6^/mouse). Peripheral parasitemia given as percent of RBC that are infected (**A**), hemoglobin levels (g/dl) (**B**), clinical symptoms measured by evaluating motor abilities based on a 10 point scale (higher scores correspond more advanced disease) outlined in methods (**C**), and survival (**D**) are shown for each group with time (days) post infection. Each circle in (**A**–**C**) represents an individual mouse and lines represent mean values. Data is representative of three independent experiments each carried out with at least 10 mice per group.

### *Pb*NK65^F^*does not induce ECM*

We first compared infections of C57BL/6 mice with the mutant *Pb*NK65^F^ parasite to infections with ECM-inducing WT *Pb*A and non-ECM-inducing WT *Pb*NK65^S^. Mice were inoculated with 10^6^ iRBCs. As expected, WT *Pb*ANKA^F^ infections resulted in relatively low parasitemia levels over seven days, with rapid reductions in hemoglobin levels to 8 g/dl, worsening clinical symptoms and 100% mortality by day 7 post infection (d7p.i.) (Fig. 1). In contrast, infection with WT-*Pb*NK65^S^ showed increases in parasitemia reaching 40% by d20p.i., with a decrease in hemoglobin levels to 2 g/dl, worsening clinical symptoms beginning around d15p.i. and 100% mortality by d25p.i. This disease progression was nearly identical for mice infected with the mutant *Pb*NK65^F^ indicating that the AP2^F^ mutation in *Pb*NK65 did not alter the course of the non-CM infection of WT-*Pb*NK65^S^ to resemble infections by the CM-causing WT-*Pb*ANKA^F^.

**Figure 2:**
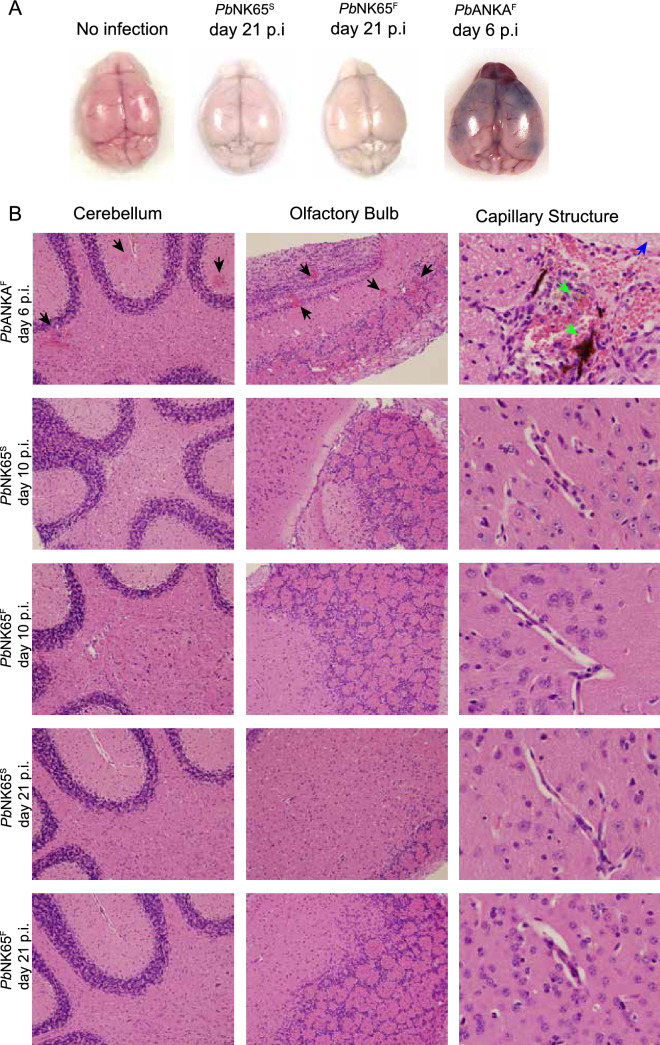
S1823F mutation in ApiAP2 TF of *Pb*NK65 does not induce experimental cerebral malaria. (**A**, **B**) Mice were inoculated with WT *Pb*NK65^S^, mutant *Pb*NK65^F^ or WT *Pb*ANKA^F^ iRBCs as in Fig. 1 and brains were collected from infected mice and uninfected controls at various time points. Sites of brain hemorrhage are shown as patchy dark blue colorations on brain photos of Evans blue injected mice. Photos are representative of more than 10 mice per group (**A**). (**B**) Tissue sections taken from different parts of infected mouse brains were stained with Hematoxylin and Eosin and then evaluated under light microscopy for histological signs of brain pathology. Sites of hemorrhage in 10 × magnified slides are shown with black arrow heads. RBC congested areas and signs of edema are shown in 40 × magnified slides with green and blue arrow heads respectively. 3 mice/group/day were used for collecting tissue sections.

ECM is accompanied by loss in BBB function by d6 p.i. as measured by leakage of Evans blue dye into the brain^11,12^. To determine if *Pb*NK65^F^ induced cerebral pathology, we examined the brains of mice euthanized shortly after Evans blue was administered intravenously either at d6 p.i. for WT *Pb*ANKA^F^ or d21 p.i. for WT *Pb*NK65^S^ and *Pb*NK65^F^ (Fig. 2A). As expected, brains from *Pb*ANKA^F^ -infected animals showed loss of BBB integrity predominantly in the olfactory bulbs of the brain. In contrast, the brains of WT *Pb*NK65^S^- and *Pb*NK65^F^-infected mice appeared similar to brains of uninfected control animals albeit paler, likely related to their severe anemic states. Histopathological evaluation of brain sections showed that signs indicative of ECM including hemorrhage in the cerebellum and olfactory bulb, and iRBC sequestration in the brain vasculature, were only observed in WT *Pb*ANKA^F^-infected mice but not in *Pb*NK65^F^ or WT *Pb*NK65^S^-infected mice (Fig. 2B).

**Figure 3:**
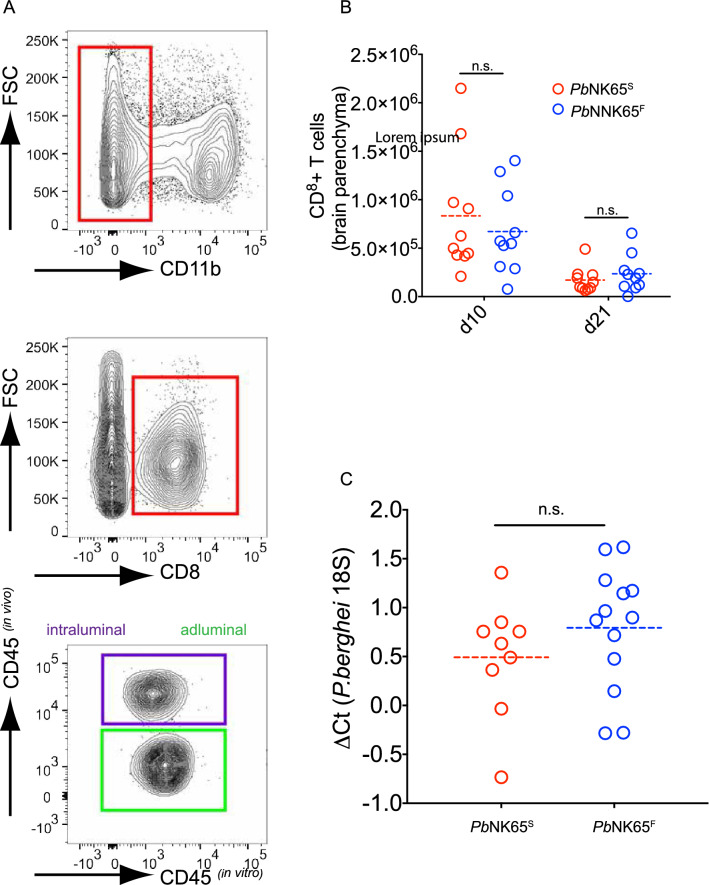
*Pb*NK65^F^- and *Pb*NK65^S^-infected mice have similar quantities of CD8^+^ T cells and iRBCs in their brains. (**A**, **B**) Mice were infected as indicated in Fig. 1. At days 10 or 21 post infection mice were injected intravenously with AF-488 conjugated CD45 specific Abs (in vivo) to label intravascular leukocytes. Brains were harvested shortly after and processed as detailed in methods section. Lymphocytes were then incubated with a second Ab including BV421 labelled CD45 specific Abs (in vitro) which stains both vascular and extravascular leukocytes. CD11b was used to gate out microglia and CD8 was used to gate CD8^+^ T cells. Within the CD8^+^ T cell gate cells that are double positive for both CD45 stains are gated as intraluminal cells (purple gate) and cells that are single positive for only in vitro CD45 stain were gated as adluminal cells (green gate) (**A**). Absolute counts of parenchymal CD8^+^ T cells are graphed (**B**). Each circle represents an individual mouse n.s. = P > 0.05 (One way ANOVA with Dunnet’s multiple comparisons test). (**C**) qPCR comparisons of parasite levels in brain specimens taken at day 21 post infection from mice infected as indicated in Fig. 1. Each circle represents an individual mouse. n.s. = P > 0.05 (Welch’s T-test).

ECM is also accompanied by accumulation of CD8^+^ T cells in the brain^6,11,12^. We quantified the CD8^+^ T cell accumulation in single cell preparation of the brains of WT *Pb*NK65^S^- and mutant *Pb*NK65^F^-infected mice using a double leukocyte staining strategy detailed in the methods section. We observed no difference in the quantities of CD8^+^ T cells between *Pb*NK65^S^- and *Pb*NK65^F^-infected mice (Fig. 3A,B) at either d10 p.i. or d21 p.i. We quantified iRBC sequestration in brains, another sign of ECM patology^11,12^ using qPCR and observed no differences between *Pb*NK65^F^- and *Pb*NK65^S^-infected mice (Fig. 3C). Taken together these results demonstrate that the introduction of an S → F substitution in *Pb*NK65 is not sufficient to convert a non-ECM inducing parasite into an ECM generating parasite.

### ***PbANKA***^***S***^*** induces ECM***

**Figure 4:**
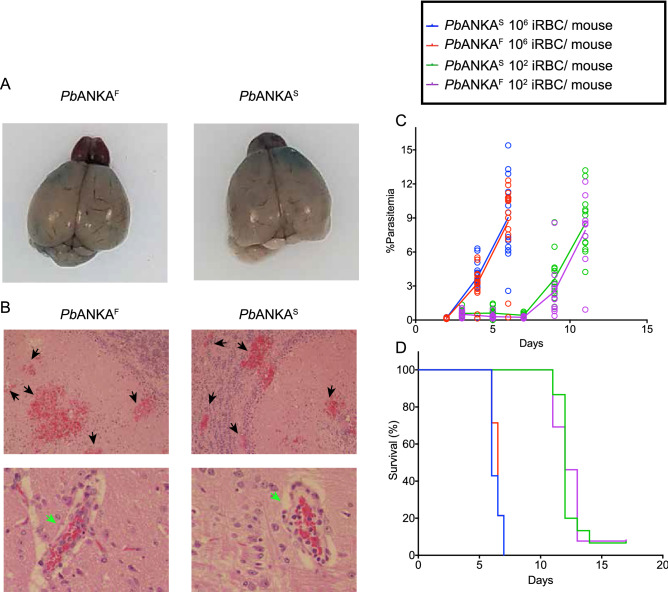
Mutating the ApiAP2 TF of *Pb*ANKA to ApiAP2 of *Pb*NK65 does not alter its ECM inducing ability. (**A**, **B**) Mice were infected with 10^6^ iRBC/mouse dose of mutant *Pb*ANKA^S^ and WT *Pb*ANKA^F^ parasites microanatomic changes showing dark colored hemorrhagic areas in Evans blue stained brains (**A**) and histologic changes showing hemorrhagic areas (black arrow heads) and RBC congested capillaries (green arrow heads) in hematoxylin and Eosin stained brain sections are shown (**B**). (**C**, **D**) Two different inoculation doses were used to infect mice and changes in parasitemia (**C**) and survival (**D**) are graphed. Each circle represents a single mouse and line represents mean for (**C**) Data is representative of two independent experiments.

Thus far our investigations revealed that S1823F mutation in the DNA binding domain of ApiAP2 TF PBANKA_0112100 of non-ECM generating rodent *Plasmodium* strain *Pb*NK65 was not sufficient to induce ECM in infected mice. However, it is possible that AP2^F^, although not sufficient to induce ECM when expressed on genetic background of a non-ECM inducing parasite, might be necessary for ECM induction in WT-*Pb*ANKA-infected mice. We evaluated symptoms of ECM in mice infected with 10^6^ WT-*Pb*ANKA^F^ iRBCs with the mutant *Pb*ANKA^S^ iRBCs. Evans blue staining showed comparable BBB dysfunction at d6 p.i. (Fig. 4A) and histopathological evaluation of brain sections confirmed cerebral hemorrhages and intravasculature sequestrations of iRBCs (Fig. 4B). Thus, F at position 1823 in ApiAP2 does not appear necessary to induce ECM. We also determined the outcome of infections at a lower inoculation dose of iRBCs (10^2^ versus 10^6^ iRBCs/mouse). We observed a delayed onset of the rise in parasitemia in mice receiving lower inoculum (Fig. 4C), however, the parasitemia reach comparable levels (~ 8%) (on d5p.i.) for mice given the high dose of iRBCs and (on d11p.i.) for mice given the low dose of iRBCs (Fig. 4C). Moreover, nearly 100% of mice died on d7 p.i. for mice given the high dose iRBCs or on d15 p.i. for mice given the low dose of iRBCs (Fig. 4D).

## Discussion

CM is a deadly disease that not only claims the lives of African children each year but also leaves approximately 25% of survivors with severe, debilitating neurological sequelae. At present there are no adjunctive therapies that given with anti-malaria drugs reduce CM mortality, that remains high at 15–25%. Clearly, a better understanding of the parasite genes that are necessary and/or sufficient for CM would benefit a search for new therapies. Here we investigated the contribution of a SNP that encodes amino acid 1823 in the TF ApiAP2 and differs between *Pb*A parasites that cause CM and the closely related *Pb*NK65 parasites that do not cause CM. This SNP was of particular interest as it is one of only 21 SNPs that differ between *Pb*ANKA and *Pb*NK65.

Parasites deficient for PBANKA_0112100 cannot be generated due to the vital role of ApiAP2 in the blood stage infection^21^. However, we recently showed that the SNP leading to S to F nonsynonymous amino acid substation at position 1823 of PBANKA_0112100 resulted in alteration of DNA binding site which resulted in the differential expression of 46 *Pb* genes, most of which were predicted to play a role in host pathogen interaction and immune evasion strategies of the parasite. We showed that as compared to infections of mice with WT *Pb*NK65 that resulted in the death of 100% of mice, infection with *Pb*NK65^F^ resulted in an early IFN-γ response and expansion of germinal centers leading to high levels of protective iRBC-specific TH1-type IgG2b and IgG2c antibodies. Thus, *Pb* ApiAP2 functioned as a critical parasite virulence factor in *Pb* infections^22^. Whether or not this SNP altered the progression of ECM in infected mice was not studied.

Here we report that the SNP encoding F at position 1823 of the ApiAP2 TF is neither necessary for induction of ECM by *Pb*ANKA parasites nor sufficient for the induction of ECM by *Pb*NK65 parasites. Detailed survival, parasitemia, clinical scoring, brain histopathology and sequestration analyses showed that the introduction of the F → S mutation in *Pb*A-AP2 did not alter its ability to induce CM. Similar analyses showed that introduction of an S → F mutation in *Pb*NK65 failed to generate a parasite capable of inducing ECM. Using a lower inoculum of the mutant and WT *Pb*ANKA, we showed that a 10,000-fold decrease in inoculum, although delaying the onset of the ECM symptoms, did not affect the disease outcome.

Based on these observations we conclude that the polymorphism in PBANKA_0112100 is neither sufficient nor necessary for induction of ECM. CM may result from complex interactions between different parasite genes. If this is the case mutations of multiple genes might be required to identify genetic contributions to the ECM phenotype, a costly and time-consuming process. However, further studies may reveal the functions of the currently uncharacterized 20 genes that differ between *P. berghei* NK65 and *P. berghei* ANKA and together, along with a thorough transcriptome analysis of these two parasites, may lead to identification of genetic variations that are collectively playing a role in the induction of ECM. Finally, noncoding elements of parasite genome may be exerting regulatory functions that collectively lead to a gene expression profile that would collectively lead to CM pheonotype. This possibility may only be addressed by in depth studies that are aimed towards identifying the differences between *P. berghei* NK65 and *P. berghei* ANKA in the noncoding parts of their genome and also having functional characterizations of noncoding regulatory elements.

## Methods

### Animals and *Plasmodium* strains

For all experiments, WT 8 weeks old C57BL/6 female mice, purchased from Jackson Laboratories were used. Mice were maintained in NIAID animal facilities according to Animal Care and Use Committee guidelines. Experiments involving mice were approved by IACUC. WT *Plasmodium berghei* NK65 (NYU) (*Pb*NK65^S^) and WT *Plasmodium berghei* ANKA (*Pb*ANKA^F^) strains were used for infections. *Pb*NK65^F^ mutant parasite was from stocks generated earlier for a previous project which had been confirmed as free of off-site mutations^22^. Mutant *Pb*A-AP2^S^ was generated by employing a similar mutation and cloning strategies as has been described earlier^22^.

### Construction of plasmid for editing AP2 (PBANKA_0112100) in *P. berghei* ANKA

For editing the AP2 (PBANKA_0112100) gene in *Plasmodium berghei* ANKA, replacing phenylalanine 1823 with serine (F1823S), we used the clustered regularly interspaced short palindromic repeat (CRISPR)-CRISPR-associated protein 9 (Cas9) system (CRISPR/Cas9)^23^. A single plasmid system containing pYC plasmid express all the essential components of CRISPR-Cas9; Cas9 endonuclease expressed as a fusion protein with drug selection marker hDHFR, gRNA:tracrRNA chimera driven by PyU6 promoter and a homology template for repair of double stranded break and concomitant introduction of desired mutation. A guide sequence of 20 nucleotides (5′ GCTGAATTAAAACCCCAAAG 3′), with protospacer adjacent motif (5′ AGG 3′), was selected by manual curation for targeting the Cas9 endonuclease to result in the desired editing (5,467 TTT to TCT) in the AP2 gene. The 900 nucleotides of synthetic sequence (given in Supplementary Information) containing the mutated guide region and the desired single nucleotide polymorphism (SNP) (5,467 TTT to TCT) and shield mutations to overcome repeated restriction of the modified genomic locus, was sub-cloned in the pYC plasmid using NcoI and XhoI restriction enzyme sites. The resulting plasmid, pYC_ANKAAP2NK65, was used for the transfection of the *P*. *berghei* ANKA parasites. The *P. berghei* ANKA parasites were transfected with the plasmid pYC_ANKAAP2NK65 as described earlier Successful editing of AP2 gene (PBANKA_0112100) in *P. berghei* ANKA was confirmed by DNA sequencing using the PCR strategy as described earlier^22,24,25^. Briefly, oligos (AP2F: 5′ GATTATAGATACAAATAATGAGAAAATGGG 3′ and AP2R: 5′ GCATATGTGATAGTGTTATTTCCATC 3′) corresponding to ApiAP2 that were outside the boundary of the 900 bp homology template were used to PCR amplify the region of interest from transgenic PbANKA^S^ genomic sequence. The amplicon was DNA sequenced to verify the CRISPR mediated gene editing.

### Infection of mice with infected red blood cells

Frozen stocks of parasite infected red blood cells were thawed and injected to donor C57BL/6 mice. Parasite levels in donor mice blood was checked routinely using flow cytometry-based strategies complemented with blood smear analysis as described previously^12^. Once parasitemia reached around 5–10% donor blood was taken and then diluted to final concentration: 10^6^ infected RBCs/mice for standard-dose inoculation and 10^2^ infected RBCs/mice for low-dose inoculation using sterile PBS. Mice were infected with the desired inoculation dose through a single intraperitoneal injection of 200 µl infected RBC solution.

### Analysis of disease progression

In order to analyze disease progression in experimental groups of parasite infected animals, blood samples were taken routinely and parasitemia was measured using flow cytometry and/or smear, hemoglobin levels were measured using HemoCue Hb201 analyzer (HemoCue, Brea, CA, USA). Disease related worsening in motor abilities and general condition were assayed using 10-point clinical scoring system that consists of 1-gait/posture/appearance, 2-cage grasp, 3-interactions/reflex, 4-visual placing, and 5-capacity to hold their body weight on a baton. Each subcategory was scored 0 (healthy/normal), 1(moderate incapacitation) or 2 (total loss of ability)^11,12^. Mice were evaluated and scored by trained personnel in a single blind fashion routinely. A combined clinical score of 6 or above and hemoglobin levels of 2.5 g/dl or below were considered as end-point criteria and once animals reach either one of these criteria they were euthanized. Changes in the levels of parasite infected red blood cells (parasitemia) were monitored by routine blood analyses using a flow cytometry-based analysis developed earlier^12,26^. These findings were later confirmed using blood smears prepared from the same samples.

### Assessment of brain pathology

The integrity of blood brain barrier was evaluated by injecting the mice with 20 mg/kg Evans blue 3 h before euthanasia. Brains were then removed, and pictures were taken. To visualize histopathological changes in brain, tissue sections obtained from different parts of the brain were stained with hematoxylin and eosin as described previously and evaluated under light microscope at 10 × to 40 × magnification^11,12^.

To compare parasites localized in brain parenchyma, anesthesized mice were performed intracardiac perfusion, followed by euthanasia and removal of brains. Brains were immediately frozen in liquid nitrogen and kept at − 80 °C until processing. Brains were then homogenized and RNA was isolated using Qiagen RNeasy Plus mini kit according to manufacturer’s guidelines. cDNA synthesis was carried out using BioRAD iScript cDNA synthesis kit. SYBR green PCR master mix (Bio-Rad) was used to amplify 18S rRNA as well as host control genes, hprt, gapdh, and ppia. The primer sequences used are as follows *Pb*-18S: 5′-AAGCATTAAATAAAGCGAATACATCCTTAC-3′ and 5′-GGAGATTGGTTTTGACGTTTATGTG-3′. The mouse *hprt*: 5′-TGCTCGAGATGTGATGAAGG-3′ and 5′-TCCCCTGTTGACTGGTCATT-3′, mouse *gapdh*: 5′-GTGGAGTCATACTGGAACATGTAG-3′ and 5′-AATGGTGAAGGTCGGTGTG-3′, mouse *ppia*: 5′-TTCACCTTCCCAAAGACCAC-3′ and 5′-CAAACACAAACGGTTCCCAG-3′, Geometric means of threshold cycle (*C*_*T*_) values of all three control genes were subtracted from the *C*_*T*_ value of 18S rRNA gene and thus Δ*C*_*T*_ values were obtained. Comparing these values between WT and mutant parasite infected brains was used to interpret any changes in parasite loads in the brains.

To distinguish circulating CD8^+^ T cells and CD8^+^ T cells that were recruited to brain parenchyma, parasite infected C57BL/6 mice were injected in tail vein with 200 µl PBS solution containing 12 µl AF488 conjugated anti mouse CD45.2 (Biolegend, Catalog No: 109816). Fluorescently labelled antibody was allowed to circulate for 2 min which resulted in labeling of all intraluminal leukocytes. Mice were then immediately euthanized, and brains were removed. Lymphocytes from brain were harvested as previously described^11,27^. Single cell suspensions of lymphocytes were stained with Live/DEAD Near IR (Thermofisher) viability dye as well as BV785 conjugated anti CD8 (to label CD8^+^ T cells), BV605 conjugated anti CD11b (to gate out microglia) and BV421 conjugated anti mouse CD45.2 to label all leukocytes. Flow cytometry analysis was carried out using BD LSR II and data was analyzed using Flowjo software. CD8^+^ T cells that were stained with both BV421 and AF488 were from the intravascular pool while CD8^+^ T cells that were stained only with BV421 but not the intravascularly injected AF488 were from the adluminal pool.

### Ethics statement

This study has no human participants. Animal experiments were carried out according to ACUC guidelines and NIH approved animal protocol: ASP No:LIG-2E.

## Supplementary information

Supplementary Information.

## Data Availability

All data supporting these results are available from the corresponding authors upon request.
