# Supplementary material for: Testing the impact of a single nucleotide polymorphism in a Plasmodium berghei ApiAP2 transcription factor on experimental cerebral malaria in mice
Source: Sci Rep. 2020 Aug 12;10:13630. doi: 10.1038/s41598-020-70617-7 (PMC7424516; doi:10.1038/s41598-020-70617-7)
Supplement: Supplementary file 1 — Supplementary Information. [file 41598_2020_70617_MOESM1_ESM.pdf]

Supplementary Material

Testing the impact of a single nucleotide polymorphism in a *Plasmodium berghei* ApiAP2 transcription factor on experimental cerebral malaria in mice

Munir Akkaya[1,#,*], Abhisheka Bansal[2,#,++,], Patrick W. Sheehan[1,#+] , Mirna Pena[1], Clare K. Cimperman[1,+++], Chen Feng Qi[1], Takele Yazew[1,++++], Thomas D. Otto[3], Oliver Billker[4], Louis H. Miller[2], Susan K. Pierce[1,*]

[1]Laboratory of Immunogenetics, National Institute of Allergy and Infectious Diseases, National Institutes of Health, Rockville, Maryland; [2]Laboratory of Malaria and Vector Research, National Institute of Allergy and Infectious Diseases, National Institutes of Health, Rockville, Maryland; [3]Institute of Infection, Immunity and Inflammation, University of Glasgow, Glasgow UK. [4]Laboratory for Molecular Infection Medicine Sweden and Molecular Biology Department, Umea University, Umea, Sweden

+Current address: Department of Neurology, Washington University School of Medicine in St. Louis, St. Louis, MO

++Current address: Jawaharlal Nehru University, School of Life Sciences, New Delhi, India

+++Current address: Department of Microbiology University of Pennsylvania, Philadelphia, PA

++++ Current address: Department of Veterinary Medicine, University of Maryland, College of Agriculture and Natural Resources, College Park, MD

[#]MA, AB and PWS contributed equally to this work

*Address correspondence to Munir Akkaya (munir.akkaya@nih.gov) or Susan K. Pierce (spierce@niaid.nih.gov) Laboratory of Immunogenetics, 5625 Fishers Lane, Room 4S04, Rockville, MD 20852 USA

Supplementary Figure 1 Generation of *Plasmodium berghei* ANKA with NK65NYU AP2 (ANKA_011210), (*Pb*ANKAs). (A) Diagrammatic representation of the strategy for generating transgenic ANKA (AP2NK65NYU) strain. The CRISPR-Cas9 gene editing tool was used to substitute TTT in the WT ANKA (target) to TCT in ANKA AP2NK65NYU (modified) resulting in change of phenylalanine to serine. The gene sequence of AP2 in ANKA (ANKA_011210) from 5428–5469 nucleotides, highlighting the guide region (20 nucleotides) and the protospacer adjacent motif (PAM) are shown. The modified sequence shows the mutated nucleotides (bold) called 'shield mutations' that prevent re-cutting of the modified locus. (B). Confirmation of AP2 gene editing in ANKA AP2NK65NYU. The figure shows the region of AP2 from 1809-1823 amino acids and the corresponding DNA sequence for the ANKA (WT) and ANKA (AP2NK65NYU). The corresponding chromatogram is shown.

Supplementary Figure 1

A

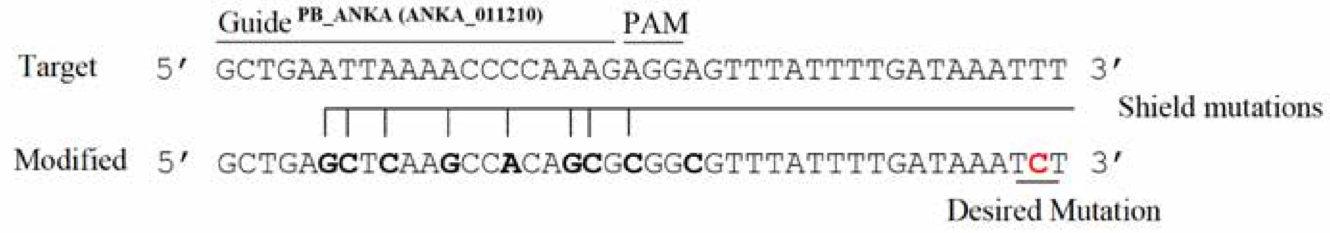

B

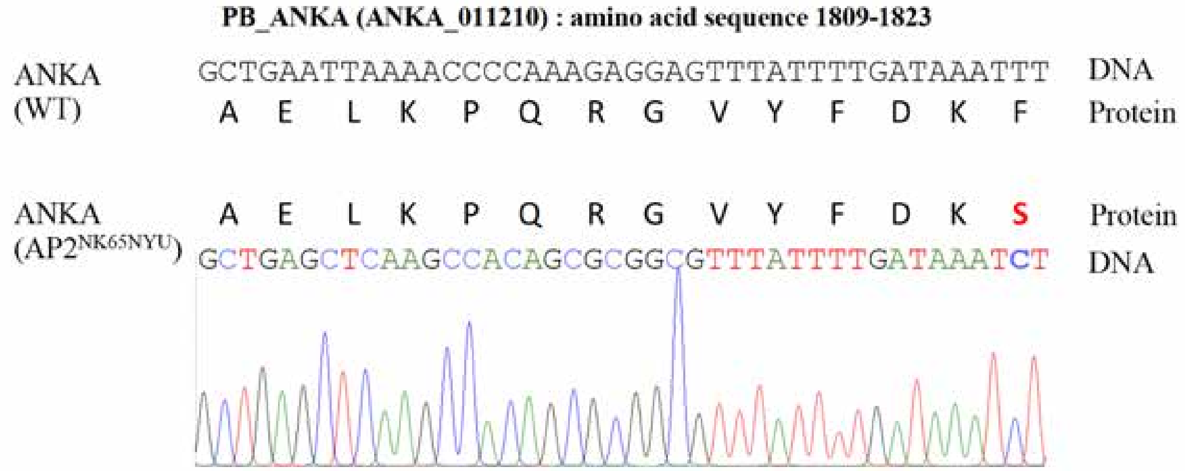

Supplementary Information

```
ATTTGAATGAAGGGGAACATAAAGAAAATAATGAACCTCACAGAAATACAGATAATCAAAAAATCGGAATAAAAGTA
GATAAAAATGGTAAAAATGGAAATATATCTATGAACGGTTTATTTGTAAGTTATGGACGTGGACAAAGGACAAAAGg
taaataaaaaatgttgaatgctaaatattattgaaaataaacaagcgttatattttattattttactttttatagGA
TTAGAAAAAAATAAAAACTCAGCGGACAATGCAAGTTCGAATAAGAATAGTGGAAACACAAAAGGAAATGGATCTAA
AAGAGGCAATGGAAAAGATAAACAAAATGGTGGATGTTATGTAGACATTGGTGAAAATTATGAACTTAAATATACAG
TTGCTGAGCTCAAGCCACAGCGCGGCGTTTATTTTGATAAATCTCAAAAAGCATGGATAGGAAGTTGGTATGAAGAA
GGCAAACAAATAAAACGTCGATTTAAAATTAAATATTATGGTTGGGACGAAGCTAAAGAATTAGCAACAAAAGCTAG
ATTTTCTTTTGAAAATCGAATCAAAAATTTAGAAGGAAATAACGGAAAAGGTAATTCTAGTACATCTAAAAATAATA
CAAATTCAGGAGAAAAGAATGGAACAAAACTTACAGTAGTTAATAATAAAAAAAGTGGAATTCAGGATATAGAAAAT
AATAATAATGATAGAATAAAAACAAGAAACAGTTCAAAAGATGTAGAAGAAAAAAATAATAATGAAAATGCTGGATT
TGATGAAAATAATTTTAATGATATAGATAATCATAATAATCCAAATGAAAAAAATAATTCTGATGATAAAAAAAAAA
ATAATAATATAGATGATGAAATATGGAGTAATCAAGAATCTCAAAATTGTGAT
```

A 900 bp homology region was used to incorporate the desired substitution ($AP2_{F/S}$) in the AP2 gene (PBANKA_011210) in *P. berghei* ANKA strain by altering TTT to TCT (‘C’ highlighted in red). The DNA sequence of the guide region (underlined) was altered with synonymous mutations to avoid recognition of the edited locus by Cas9 endonuclease.
